# High dose nitroglycerin treatment in a patient with cardiac arrest: a case report

**DOI:** 10.4076/1752-1947-3-8782

**Published:** 2009-08-10

**Authors:** Maya Guglin, Gilbert Postler

**Affiliations:** 1 Department of Cardiology, University of South Florida, 2 Tampa General Circle, Tampa, FL 33606, USA

## Abstract

**Introduction:**

Vasodilators like nitroglycerin or nitroprusside improve hemodynamics in patients with advanced heart failure. However, using these agents in critical conditions is limited because of their ability to decrease systemic blood pressure.

**Case presentation:**

We report the rapid effect of high dose intravenous nitroglycerin treatment in an 86-year-old man after cardiac arrest and prolonged resuscitation, together with previous observations and a brief review of the literature.

**Conclusion:**

High dose intravenous nitroglycerin can be beneficial in cardiac arrest.

## Introduction

Vasodilators like nitroglycerin (NTG) or nitroprusside improve hemodynamics in patients with advanced heart failure (HF). However, using these agents in critical conditions is limited because of their ability to decrease systemic blood pressure (BP). We report a case of successful use of high-dose intravenous NTG in a patient after cardiac arrest.

## Case presentation

An 86-year-old Caucasian man with a history of coronary artery disease, coronary artery bypass surgery in 1999, hyperlipidemia and hypertension, came to the out-patient laboratory of the hospital to check the results of his blood tests. His home medications included 40 mg daily of lisinopril, 40 mg daily of furosemide, and 80 mg daily of simvastatin. He complained of sudden pressure-like chest discomfort and collapsed in the waiting area. The "code blue" emergency call was given. The team responding to the call applied an automated external defibrillator and delivered a shock. The recorded rhythm indicated ventricular tachycardia. Consequently, the patient was given 300 mg of amiodarone, which resulted in the restoration of his normal sinus rhythm. The ventricular tachycardia was immediately restarted. For the next 16 minutes, ventricular tachycardia and ventricular fibrillation were treated with additional shocks and multiple doses of epinephrine, amiodarone and lidocaine.

After 16 minutes of resuscitation, the patient's normal sinus rhythm was restored, and he was transferred to the medical intensive care unit, intubated and started on mechanical ventilation. He was unconscious, and his BP was 98/58. The laboratory tests drawn at this time showed his white blood cells at 16.6 G/L, hemoglobin 10.2 G/dL and platelets 175 G/L. His coagulation test results and chemistry were normal.

After 15 minutes of normal rhythm, the patient again developed ventricular tachycardia. Following the advanced cardiac life support protocol, resuscitation efforts continued for 50 more minutes, using epinephrine, vasopressin, amiodarone, lidocaine, magnesium and bicarbonate. Eventually, the monitor recorded asystole and the patient had no spontaneous respirations, pulse, BP, or heart sounds. Based on previous experience with a patient, we gave him 4 mg of NTG intravenously as a bolus as a last resort. After three minutes of chest compressions the patient was pronounced dead and all resuscitative efforts were stopped.

Two minutes later, however, the nurse discovered that the patient was breathing, in normal sinus rhythm, with a palpable pulse, and a BP of 137/58 mmHg. The ventilator was turned back on, and the patient was treated with NTG, heparin, amiodarone drips and aspirin.

The next morning, the patient's troponin I level was elevated at 23.9 ng/mL (upper normal limit 0.05), which is consistent with acute myocardial infarction (MI). His electrocardiogram displayed normal sinus rhythm and his left bundle branch block was the same as pre-event recordings. His chest X-ray was negative. He was extubated on the second hospital day, with his mental status gradually returning to his baseline. His BP remained stable at 120-150 mmHg systolic. An echo displayed a reduced ejection fraction of 30% with global hypokinesis and an akinetic inferior wall. An adenosine-sestamibi stress test displayed areas of infarction of the inferolateral and anterolateral walls with a small area of reversibility. Taking his advanced age into account, we decided against performing cardiac catheterization. He subsequently underwent implantation of an automated defibrillator and was discharged from the hospital on day 18.

## Discussion

Only one known similar case has been previously reported in the English language literature. In 1984, Ward and Reid [1] described a 64-year-old woman with an acute MI who had a cardiac arrest. She had ventricular tachycardia and was cardioverted to sinus bradycardia, intubated and ventilated. She received atropine, epinephrine, and calcium chloride, and had pericardiocentesis and rapid saline infusions. However, she still did not have a detectable BP. She was then started on NTG at 1 mg/minute. In three minutes, her BP was 80/60 mmHg. Several days later, her cardiac catheterization demonstrated severe coronary artery disease and a large anteroapical and septal infarction with aneurysm formation. She was eventually discharged from the hospital.

In 1990 [2], and in 1997 [3], we reported a case series in which the infusion of high doses of NTG resulted in rapid improvement in some patients with cardiogenic shock due to acute MI or advanced heart failure. The results are summarized in Table 1.

**Table 1:** BP Response and Clinical Outcomes after High Dose NTG

S. No	Sex	Age	BP, mm Hg		NTG, m	Time, min	Outcome
							
1	m	59	0	80/60	20	30	died
2	f	77	0	0	5	5	died
3	m	60	0	0	15	10	died
4	f	62	0	40/30	48	20	died
5	f	63	0	40/30	10	1	died
6	f	63	0	140/90	10	5	died
7	m	43	0	80/70	6	2	died
8	m	68	0	80/60	15	10	died
9	m	63	0	80/60	20	10	died
10	m	54	0	80/60	10	10	survived
11	f	71	0	90/70	35	15	survived
12	f	65	80/70	100/80	15	10	survived
13	m	78	0	120/80	48	15	survived
14	m	62	0	140/80	40	10	survived
15	m	60	0	90/70	25	3	survived
16	f	52	60/50	80/60	15	10	survived
17	f	64	0	120/80	5	1	survived
18	m	64	0	80/60	40	5	survived
19	m	57	80/60	100/60	1	50	survived
20	m	67	60/40	110/70	10	2	survived
21	m	67	0	100/60	30	5	survived
22	m	47	0	80/60	25	10	survived

High doses of NTG were used in 22 patients, including 14 patients with acute MI and eight patients with advanced HF. All patients had critically low BP measured by cuff, and 18 had an unmeasurable BP and pulse. They all had cold and mottled skin and increased central venous pressure. Eleven patients had rales in the lungs, three had pulmonary edema, and one had anasarca. The doses of NTG used in each patient, as well as the times of infusions, are listed in Table 1. BP became obtainable or increased in 20 of 22 patients immediately after intravenous NTG was administered. In the end, 13 patients survived.

We recently presented these data to a group of cardiology fellows, one of whom administered the bolus of NTG to our patient in this case.

According to current recommendations, intravenous NTG is contraindicated if systolic BP is below 90 mmHg. Hemodynamic properties of vasodilators, and of nitrates in particular, were extensively studied in the 1970s and 1980s, although usually not in terminal patients with no BP. In 1972, Franciosa et al. [4] reported that intravenous sodium nitroprusside increased cardiac output and decreased wedge pressure in 15 patients with acute MI and elevated left ventricular filling pressure. Their BP was not allowed to fall below 95 mmHg, with the average drop in systolic BP at only 26 mmHg. Similar results were achieved in severe HF secondary to ischemic or dilated cardiomyopathy [5].

In 1975, Chatterjee et al. [6] described a beneficial effect of nitroprusside in 43 patients with acute MI and severe pump failure. In their series, the cardiac index increased from 1.7 to 2.2 L/min/m^2^, while the left ventricular filling pressure decreased by 35%. The mean arterial pressure decreased from 83 ± 1.5 to 73 ± 1.7 mmHg. Although these patients had BP of ≤90 mm Hg by cuff, only 17 had clinical shock syndrome.

In another study evaluating incremental doses of intravenous NTG in patients with left ventricular failure the maximal hemodynamic benefit, in terms of decrease in wedge pressure and increase in cardiac index, was obtained at 160 mcg/min, which represented the highest dose tested [7].

Stevenson et al. [8] found that after-load reduction with nitroprusside in severe HF leads to smaller left ventricular end-diastolic volume and less severe mitral regurgitation, resulting in greater forward flow. The BP cutoff for nitroprusside infusion was 80 mmHg. In the emergency department, boluses of intravenous NTG ranging from 0.05 mg to 0.4 mg repeated every five minutes as needed for chest pain were used successfully to treat ischemia due to MI or unstable angina. Systolic BP was not allowed to drop below 95 mmHg [9].

Recently, there has been increased interest in high dose intravenous nitrates. Cotter et al. [10] randomized patients with pulmonary edema into cohorts receiving isosorbide dinitrate at 3 mg bolus administered intravenously every five minutes versus traditional treatment using low doses of isosorbide, furosemide, and morphine. BP was not allowed to be <90 mmHg. Mechanical ventilation was required in 13% of the high dose nitrates group and in 40% of the traditional group. MI occurred in 17% and 37%, respectively. Similar results were obtained by Phillip Levy et al. [11], who administered up to 10 doses of NTG in intravenous boluses of 2 mg every three minutes to treat pulmonary edema with hypertension (systolic BP > 160 mmHg). In comparison with historical controls, fewer intubations, MIs, and intensive care unit admissions occurred.

One animal study has demonstrated the benefits of NTG in pigs after prolonged resuscitation. After four minutes of ventricular fibrillation and four minutes of standard CPR, pigs were randomized to the combination of epinephrine, vasopressin and NTG (7.5 mcg/kg) versus epinephrine alone. The mean coronary perfusion pressures, left ventricular, and global cerebral blood flow were significantly higher in animals who received NTG as part of the therapy. Spontaneous circulation was restored in 11 of 12 animals in the NTG group, versus 6 of 12 swine after epinephrine alone (P = NS) [12].

A possible explanation for the hemodynamic benefit of NTG in our patients is increased cardiac output produced by rapid vasodilatation in a heart operating at the extreme of the Frank-Starling curve. Vasodilators in heart failure with or without acute myocardial infarction have been proven to decrease left ventricular filling pressure and systemic vascular resistance while increasing cardiac index [7]. The more severe the failure, the more beneficial the effect of vasodilators [13].

Interventricular dependence can also be a factor in cases where elevation of right ventricular pressure compromising filling of the left ventricle occurs due to severe congestion resulting from pre-existing systolic dysfunction and precipitated by ongoing ischemia. In this case, decreased preload after NTG can improve left ventricular filling and further increase cardiac output [14].

## Conclusions

In summary, we have presented a case where high dose NTG in cardiac arrest causes dramatic effects on a patient. Further investigations are needed. In particular, such treatment could be tested during the resuscitation of cardiac patients after the exhaustion and failure of current protocols.

## Abbreviations

ACLS: advanced cardiac life support; BP: blood pressure; CPR: cardio-pulmonary resuscitation; MI: myocardial infarction; NTG: nitroglycerin.

## Competing interests

The authors declare that they have no competing interests.

## Consent

Written informed consent was obtained from the patient for publication of this case report and any accompanying images. A copy of the written consent is available for review by the Editor-in-Chief of this journal.

## Authors' contributions

MG described her past experience with high dose NTG at the fellows conference. GP who was at this conference used it on a dying patient and successfully resuscitated him. GP described the case, MG edited it and wrote the literature review and contributed her old data. Both read and approved the final manuscript.
